# Estrogen Receptors and Ubiquitin Proteasome System: Mutual Regulation

**DOI:** 10.3390/biom10040500

**Published:** 2020-03-26

**Authors:** Irina V. Kondakova, Elena E. Shashova, Evgenia A. Sidenko, Tatiana M. Astakhova, Liudmila A. Zakharova, Natalia P. Sharova

**Affiliations:** 1 Cancer Research Institute, Tomsk National Research Medical Center, Russian Academy of Sciences, 5 Kooperativny Street, 634009 Tomsk, Russia; kondakova@oncology.tomsk.ru (I.V.K.); SchaschovaEE@oncology.tomsk.ru (E.E.S.); sidenkoevgeniyaaleksandrovna@gmail.com (E.A.S.); 2 Koltzov Institute of Developmental Biology, Russian Academy of Sciences, 26 Vavilov Street, 119334 Moscow, Russia; tastakhova@bk.ru (T.M.A.); l-a-zakharova@mail.ru (L.A.Z.)

**Keywords:** nuclear estrogen receptors, membrane estrogen receptors, ubiquitin proteasome system, immune proteasomes, cancer

## Abstract

This review provides information on the structure of estrogen receptors (ERs), their localization and functions in mammalian cells. Additionally, the structure of proteasomes and mechanisms of protein ubiquitination and cleavage are described. According to the modern concept, the ubiquitin proteasome system (UPS) is involved in the regulation of the activity of ERs in several ways. First, UPS performs the ubiquitination of ERs with a change in their functional activity. Second, UPS degrades ERs and their transcriptional regulators. Third, UPS affects the expression of ER genes. In addition, the opportunity of the regulation of proteasome functioning by ERs—in particular, the expression of immune proteasomes—is discussed. Understanding the complex mechanisms underlying the regulation of ERs and proteasomes has great prospects for the development of new therapeutic agents that can make a significant contribution to the treatment of diseases associated with the impaired function of these biomolecules.

## 1. General Introduction

Estrogens and their receptors play an important role in the regulation of various functions of estrogen-sensitive cells. Estrogens belong to the family of steroid hormones synthesized in the ovaries and other tissues. The most important estrogens that have estrogenic hormonal activity are 17β-estradiol, estriol and estrone. They are responsible for the maturation and maintenance of the female reproductive function, acceleration of the synthesis of RNA, DNA and protein, and the stimulation of the proliferation and differentiation of target tissues [1,2]. Estrogens have also been shown to affect other tissues. They exert neuroprotective effects [3,4] and alter the function of immune cells [5], smooth muscle cells [6], bone tissue [7], endothelium [8], and hemostasis [9]. Estrogens increase protein synthesis, regulate lipid metabolism and affect water and carbohydrate metabolism [10,11,12]. They play an important role in prenatal and postnatal development [13]. The participation of estrogens in pathology is wide-ranging, including in cardiovascular and gastrointestinal diseases [14,15,16,17] as well as cancer [18,19]. The reception of estrogens is very important for the transmission of the estrogen signal and the realization of the effects of hormones. The regulation of the content and binding activity of estrogen receptors (ERs) is carried out at transcriptional, translational and post-translational levels. The ubiquitin proteasome system (UPS) plays a significant role in the regulation of ER function. In their turn, ERs may affect the characteristics of the proteasome pool. This review describes the structure and mechanisms of action of ERs and UPS and discusses the methods of their mutual regulation.

## 2. Estrogen Receptors

There are two different subtypes of ERs, ERα and ERβ, which belong to the family of nuclear steroid hormone receptors. They are ligand-activated transcription factors and are encoded by two different genes [20]. The human ERα gene (ESR1), a large genomic segment that spans ~300 kb, is located in the q24-27 of chromosome 6 and includes eight exons that encode a full-sized 66 kDa protein, consisting of 595 amino acids. ERβ gene (ESR2), located in q22-24 of chromosome 14, spans 254 kb, with eight coding exons. ERβ protein consists of 530 amino acids with a molecular weight of 60 kDa. Both ERs have a domain structure that includes DNA binding domain (DBD), ligand binding domain (LBD), central hinge region, and two functional transcription activation domains (AF1 and AF2) [21]. Both receptor subtypes contain the homologous central DBD (96% identity) and carboxyl (C)-terminal LBD (AF2 + LBD, 53% identity). The LBDs of ERα and ERβ represent a three-layer antiparallel α-helical fold, containing 10–12 helices. At the same time, there is a difference in the amino (N)-terminal sequence (AF1, 18% identity) between the two ER subtypes. The AF1 domain is associated with the specific transcriptional activity of ERα and ERβ and with their effects on the cells and whole body. ERα stimulates cell proliferation and ERβ inhibits it [22]. ERα suppresses apoptosis and autophagy [23], and ERβ inhibits the cell viability and mediates cell death by inducing apoptosis and autophagy [24]. ERα plays a key role for sexual behavior and other interrelated behaviors of female mice, such as parental and aggressive behaviors [25], whereas ERβ may play a significant role in the establishment and maintenance of hierarchical social relationships among male mice by regulating aggressive behavior in a social status-depending manner [26].

The complexity of estrogen-stimulated cellular responses is complemented by the specifics of activation of these domains. Specific distinctions in tissue distribution have been characterized: the content of ERα is high in the uterus (endometrium), mammary glands, ovarian stromal cells, hypothalamus, skeletal muscle, adipose tissue and bone, while ERβ in these tissues seems to play a secondary role [27,28]. It has been found that ERβ is important for the transmission of 17β-estradiol signals in granulosa cells of the ovary, prostate, lungs, cardiovascular and central nervous systems. The existence of two ER subtypes and their ability to form DNA-binding dimers indicates three potential methods of estrogen signaling: through ERα or ERβ subtype in tissues and through the formation of heterodimers in tissues expressing both ERα and ERβ [21,28].

Nuclear ERs initially exist in the cytoplasm as monomers and make dimers after binding to the ligand. The formation of a dimer is important for the function of ERα, since mutations that disrupt dimerization inhibit receptor activity [21]. After the interaction of estrogens with receptors in the cytoplasm and their dimerization, the dimer complex moves to the nucleus, followed by the activation of the transcription of estrogen-regulated genes depending on the recognition of the DBD sequence [29]. These genomic sequences are commonly called estrogen response elements and are often characterized by an inverted repeat separated by three nucleotides (5′AGGTCAnnnTGACCT3′), which are typically found in the promoter or enhancer regions of genes whose transcription is regulated by estrogens [30]. In addition, the complex of 17β-estradiol and ER is able to mediate gene expression by functional interactions with transcription factors—for example, AP-1 and Sp-1—associated with their related elements on DNA [21,22].

There is a cross-relationship between ERα and other signaling pathways: ERα acts as a coregulator by interacting with other transcription factors such as AP-1, Sp1, and nuclear factor kappa B (NFκB) [31]. Besides, ERα is phosphorylated and transcriptionally activated in response to growth factors, such as epidermal growth factor and insulin-like growth factor [32]. It has been also shown that the progesterone receptor, interacting with ERα, changes the binding of ERα to chromatin and the expression of specific genes in breast cancer cells [33].

Steroid receptors are localized, not only in the nucleus and plasma membrane, but also in mitochondria, endoplasmic reticulum, and other cellular organelles [22]. The transfer of the receptor complex to mitochondria indicates the possible role of ER in the regulation of bioenergetics [34]. Mainly, ERβ is redistributed in mitochondria, which suggests that estrogen modulates mitochondrial function through the ERβ-mediated regulation of mitochondrial DNA transcription [35].

**Figure 1:**
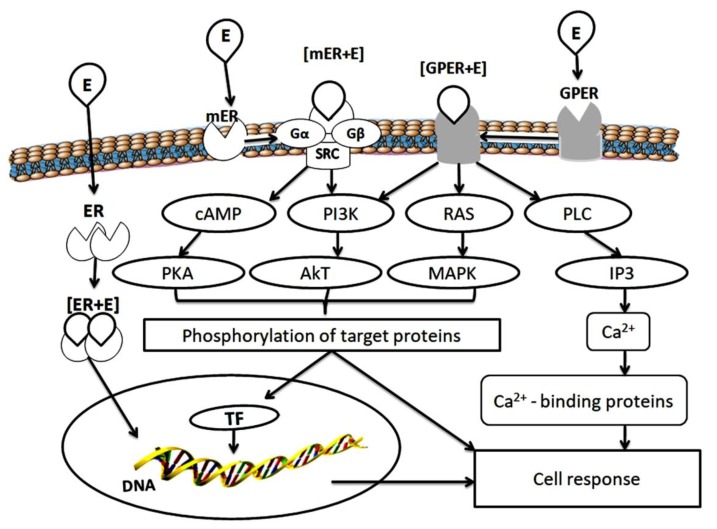
Scheme of estrogen receptor functioning. E—estrogen; ER—estrogen receptor; Gα, Gβ—G proteins; SRC—non-receptor tyrosine kinase; GPER—G protein-estrogen receptor; cAMP—cyclic adenosine monophosphate; PI3K—phosphoinositide 3-kinase; RAS—retrovirus-associated DNA sequence protein; PLC—phospholipase C; PKA—protein kinase A; Akt—protein kinase B; MAPK—mitogen-activated protein kinase; IP3—inositol triphosphate; TF—transcription factors.

In addition to the nuclear effects associated with the transcriptional activity of ER, there is a membrane-initiated steroid signaling, which provides the transcriptionally independent effects of ER. In target cells, including the pituitary, uterus, ovary, vascular epithelium, bone tissue and mammary gland, 17β-estradiol can rapidly induce ion fluxes and activate multiple protein kinases through interaction with the receptors of the plasma membrane, independent of protein synthesis [36,37] (Figure 1).

Membrane-localized receptors interact with the cellular signaling molecules on the plasma membrane to stimulate physiological responses. It has been shown that the palmitoylated monomers ERα and ERβ are transported to the membrane after interaction with caveolin-1, where they undergo dimerization upon estradiol binding. This leads to the activation of G protein subunits [36,37]. Thus, ERs can rapidly activate multiple protein kinases through signaling pathways associated with the cell membrane, including signaling pathways mediated by interaction with the G protein, independent of the activation of estrogen-dependent gene transcription (Figure 1).

Besides this, G protein-estrogen receptor (GPER) also activates the subunits of G proteins. It is recognized as the main mediator of the rapid cellular effects of estrogens [38,39,40]. GPER is characterized by the presence of seven transmembrane spirals containing the N-terminal part located outside the cell and C-terminal part in the cytoplasm. Cytoplasmic loops are involved in the selective binding and activation of various heterotrimeric proteins [40,41]. The interaction of estrogens with cytoplasmic loops of GPER activates a series of intracellular signaling events, primarily the activation of mitogen-activated protein kinases Erk1/2, PI3 kinase and phospholipase C, the release of Ca^2+^ ions affected the cell proliferation, migration, and many other processes [42,43]. Studies with the use of cell cultures and experimental animals have shown that nuclear ER and GPER are involved in the regulation of body weight, glucose and lipid homeostasis. GPER deficiency leads to increased obesity, insulin resistance, and metabolic dysfunction in mice [44].

The ER regulation occurs at various levels. The functional activity of ERα is modulated by co-activators that do not bind to DNA, but can modify chromatin (steroid receptor coactivator, SRC) [45], splicing [46], microRNA [47], long non-coding RNA [48] and post-translational modifications including phosphorylation, acetylation, ubiquitination, methylation, etc. [49] These modifications may alter the expression and stability of ER, its subcellular localization and sensitivity to hormone action. The modification of ERα by glutathione S–transferase P leads to the S-glutathionylation of cysteine, which reduces the affinity of ERα for estradiol [50].

Thus, the significance of ERs in the regulation of multiple cellular processes as well as behavioral demonstrations cannot be underestimated. In spite of ER activity being affected by numerous factors, we believe that the key role in the regulation of ER function belongs to UPS, which is a multifunctional metabolic pathway. Moreover, we offer a hypothesis of mutual regulation and cross-talk between ERs and UPS that may be involved not only in normal physiologic processes, but also in pathologic events. Before the discussion of mechanisms of ER and UPS interaction, we will consider UPS structure and functions.

## 3. Ubiquitin Proteasome System

Proteasomes, multi-subunit proteases, are generally the main actors in protein hydrolysis, and they are able to virtually target any protein [51]. The structure of proteasomes includes the 20S core and regulatory complexes [52]. Four central multidimensional coaxially laid rings of alpha and beta subunits (seven subunits in each ring) form the 20S core in the order of αββα. Beta subunits (β1, β2 and β5) have various protease activities: the β1 subunit exhibits caspase-like activity, β2 shows trypsin-like activity and β5 shows chymotrypsin-like activity. All proteolytically active centers are turned inside the proteolytic chamber of the proteasome, to prevent the accidental hydrolysis of proteins. In addition, the N-ends of the α-subunits form a hydrophobic plug, which prevents the penetration of random proteins into the proteasome. Proteasome activators contain a C-terminal hydrophobic HbYX motif that integrates into the α-ring plug and opens the entrance to the proteolytic chamber [53].

The binding of proteasome activators (19S Regulatory Complex (19S RC)**,** PA28, PA200) to α-rings contributes to a dramatic increase in proteasome activity. Small oligopeptides can undergo degradation regardless of the state of entry into the proteasome, while the functioning and state of α-rings is a key regulatory factor for the breakdown of larger polypeptides and full-sized proteins [54]. The damaged/oxidized proteins often exhibit hydrophobic structures and can be processed by the 20S proteasome alone [55]. However, this has been mostly demonstrated in vitro and the physiological mechanism by which the bulk of damaged proteins are degraded remains to be fully established.

**Figure 2:**
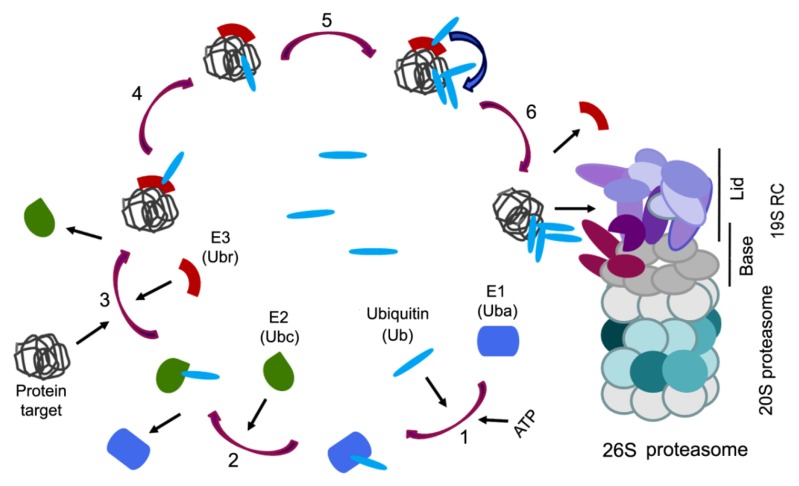
The canonical pathway of protein ubiquitination for degradation in the 26S proteasome. 1: ATP-dependent activation of the ubiquitin (Ub) by ubiquitin-activating enzyme (Uba). 2: Transfer of the activated ubiquitin to ubiquitin-conjugating enzyme (Ubc). 3: Binding of ubiquitin–protein ligase (ubiquitin-recognizing factor, Ubr), containing the homologous to E6AP carboxyl terminus (HECT) domain, to the ubiquitin and target protein. 4: Covalent attachment of the ubiquitin molecule to the target protein. 5: Building of the ubiquitin chain with the formation of isopeptide bonds Lys48-Gly76. 6: Completion of the labeling of the target protein with a structure of four (or more) ubiquitin molecules recognized by the 26S proteasome.

As a rule, proteins with a normal tertiary structure are hydrolyzed, when it is necessary, in 26S proteasomes, which include the 20S core subparticle (20S proteasome) and one or two 19S RC. For the penetration into the 26S proteasomes, target proteins are marked by a polyubiquitin chain which is formed in several stages (Figure 2).

At the initial stage, the adenosine triphosphate (ATP)-dependent activation of the ubiquitin occurs using the E1 enzyme (ubiquitin-activating enzyme, Uba), the cysteine residue of which forms a thioether bond with the C-terminal residue of glycine-76 of the ubiquitin molecule [56]. At the second stage, activated ubiquitin is transferred to the cysteine thiol group of the E2 enzyme (ubiquitin-conjugating enzyme, Ubc), the ubiquitin donor for the next stage, which can be carried out in two alternative ways. In the first case, ubiquitin is transferred to ubiquitin protein ligase (ubiquitin protein ligase or ubiquitin recognition factor, Ubr) of the E3 ligase family containing the HECT-domain. In this case, ubiquitin binds to the cysteine residue of the HECT-domain. The ubiquitin-bearing E3 ligase recognizes the target protein by the degradation signal and covalently attaches the ubiquitin molecule to the ε-NH2 group of the lysine residue of the target protein (Figure 2) [57,58]. In the second case, the E3 ligase, which does not have the HECT-domain, connects simultaneously to the target protein and E2 enzyme and orientates them close enough for allowing ubiquitination of the substrate by ubiquitin-loaded E2 [59,60]. For example, the RING E3s (the more numerous E3s) lack intrinsic enzymatic activity and act as a scaffold for the substrate and ubiquitin-loaded E2 enzyme. The combination between the E3s (>700 enzymes) and E2s (40 enzymes) allows the high specificity of targeting by UPS.

The E3 ligases can recognize the target proteins by degradation signals: sequences enriched in Pro, Glu, Ser, and Thr residues [61], N-terminal fragments containing destruction boxes [62], N-terminal residues of “destabilizing” amino acids (Arg, Lys, Leu, Phe, Asp) [63], as well as post-translational modifications (phosphorylation, joining of auxiliary proteins) [64,65]. After several cycles of ubiquitination, the protein is modified by the posttranslational addition of a ubiquitin chain and becomes detectable by the 26S proteasomes (Figure 2). Note, ubiquitin has 76 residues with seven lysines in positions, 6, 11, 27, 29, 33, 48 and 63, and each can form homotypic chains. One of the signals for the 26S proteasomes in vitro is a homotypic chain of at least four ubiquitin molecules, connected by Lys48-Gly76 isopeptide bonds between each previous and subsequent ubiquitin [66]. In cells/organs, the protein substrates carry much longer polyUb chains. Besides, homotypic chains based on Lys29, 11, 27 and 6 can target proteins for proteasomal degradation, and their formation is dependent on different stress states [67]. Lys33- and Lys63-based chains are also recognized by the proteasomes. It should be noted that some of these results were obtained in studies using cell free reconstituted systems [68]. In addition, Lys63-based polyubiquitination is required for efficient DNA repair, in a process that does not appear to involve protein degradation [69]. Heterologous chains containing more than one linkage type also exist in cells, although they represent a smaller proportion of the chains. For example, formation of a seed containing several ubiquitin molecules bound to Lys63 causes the addition of the E3 ligase that builds up the Lys48 chain. As a result, a heterogeneous branched polyubiquitin structure with mixed Lys63/Lys48 fragments is formed—the signal for degradation in the 26S proteasomes [70]. In addition, a branched polyubiquitin chain with mixed Lys11/Lys48 fragments also serves as a marker of the proteasome substrate [71,72].

The recognition of target substrates by the 26S proteasomes, and their further transmission into the proteolytic chamber, occurs due to the multi-subunit structure of the 19S RC. This regulator consists of 20–21 subunits, which can be combined into two groups: one associated with ATPase activity (Rpt subunits) and one unrelated to ATPase activity (Rpn subunits) [73,74]. The molecular mass of 19S subunits is 10–110 kDa. At least nine Rpn subunits (Rpn3, Rpn5–Rpn9, Rpn11, Rpn12, Rpn15) are part of the “lid” of the RC, which deubiquitinates the “captured” ubiquitinated proteins. Deubiquitinase sites belong to the Rpn11 subunit [75] and additional Usp14 and Uch37 subunits, which are activated upon incorporation into the 19S subcomplex [76,77,78]. The second regulator fragment, the “base” subcomplex, is formed by six Rpt subunits (Rpt1–Rpt6) and four Rpn subunits (Rpn1, Rpn2, Rpn10, Rpn13). The subunits Rpn10 and Rpn13 in this subcomplex play the role of receptors and traps of the ubiquitin chains [79,80]. In addition, the “base” subcomplex unwinds the substrate proteins due to ATP hydrolysis and opens a channel of the α-ring leading to the proteolytic chamber [81,82].

The structure of mammalian proteasomes differs not only in the presence or absence of activators, but also by a set of proteolytically active subunits. According to this feature, proteasomes can be divided into constitutive proteasomes (or “housekeeping” proteasomes) and immune proteasomes. The latter contain the subunits LMP2 (β1i), MECL1 (LMP10, β2i) and LMP7 (β5i), which are inserted into the newly formed proteasomes, respectively, instead of the constitutive subunits β1, β2 and β5 [83,84]. The conformation of substrate-binding pockets and the affinity of protein substrates to proteolytic sites are changed in the immune proteasomes [85]. As a result, chymotrypsin and trypsin-like activities (but not caspase-like activity) become more pronounced, which allows the immune proteasomes to produce several times more antigenic epitopes with the “correct” C-terminus (hydrophobic or positively charged) for the main histocompatibility complex (MHC) class I molecules, in comparison with the constitutive proteasomes. Kisselev A. et al. discovered that the length of the peptides released by the 20S proteasomes of mammalian muscle was restricted to 8-9 (instead of 3-22) amino acid residues [86]. This also can facilitate the processing by the MHC class I system.

The functions of the immune proteasomes in immune processes are not limited by the formation of antigenic epitopes for the MHC class I system. The subtype of the immune proteasomes containing the large multifunctional peptidase 2 LMP2 (but not LMP7) subunit is related to the development of the immunological tolerance to the allograft [87]. In the transplant cells, this proteasome subtype is likely to produce special peptides that inhibit the activity of cytotoxic T-lymphocytes.

The multiplicity of the proteasome forms ensures the regulation of numerous cellular processes. Proteasomes are responsible for the cleavage of growth factors, receptors, components of signaling pathways, and transcription factors [88,89]. In the next chapter, we will discuss the regulation of ER functional activity by UPS, and on the other hand, the influence of ERs on the characteristics of proteasomes.

## 4. Mutual Regulation of Estrogen Receptors and Ubiquitin Proteasome System

The first step in the interaction of ERs with UPS is ubiquitination. Currently, it is generally accepted that UPS regulates ERs in two major ways: modifying the receptors by attaching 1–2 ubiquitin molecules and thereby changing their function, and regulating their content by the cleavage in the proteasomes [90]. The monoubiquitination of ERα by ubiquitin ligases is important, both for the stability and transcriptional activity of this receptor [91]. Chain growth at the Lys48 residue leads to ER degradation in the proteasomes [92]. The ubiquitination of ERα and ERβ is known to involve the ubiquitin ligases CHIP (carboxyl terminus of Hsc70-interacting protein) and Mdm2, belonging to the class RING (really interesting new gene) [93,94,95], and ubiquitin ligase E6AP, belonging to the class HECT [96].

In addition, the ubiquitination of ERα is also associated with its phosphorylation state. Some kinases, such as Src, PKC, p38MAPK, and ERK7, have been described as ERα modifiers [31,97,98]. A key example is the sequential modification of ERα, where ER-Y537 residue is phosphorylated by Src kinase in cells treated with 17β-estradiol. The ubiquitin ligase E6AP causes the polyubiquitination of modified ERα and its subsequent degradation [97]. Thus, the phosphorylation and ubiquitination of ERα are interrelated to control both the quantity and the functions of this receptor.

In recent years, several studies have demonstrated that the inhibition of the polyubiquitination of ERα and, consequently, a decrease of its degradation by UPS led to an increase in ERα stability in cells. Protein-inhibitors of ERα polyubiquitination capable of interacting with ERα have been discovered. Inhibitors, including mucin 1, peptidyl prolyl cis/trans isomerase and others, provide its protection against ERα polyubiquitination and proteolysis [31,96]. Thus, ER cleavage in proteasomes depends on several regulatory factors.

Studies concerning the effect of proteasome inhibitors on ERα content in ER^+^ cultures of breast cancer cells showed that bortezomib, an inhibitor of chymotrypsin-like proteasome activity, decreased the proteolysis of the receptor for several hours [99]. The results obtained on the cell line of human endometrial cancer indicated a reduction in the content of ERα upon the activation of UPS [100]. It has been shown that ERβ in the prostate cancer cell culture is also cleaved in proteasomes [101]. Some ligands (synthetic ligand ICI 182,780) can promote the ubiquitination of ERs, causing their rapid degradation in proteasomes [102]. Additionally, ERα is rapidly cleaved in the proteasomes in response to the addition of 17β-estradiol to the breast cancer cell culture [99]. Moreover, chimeric molecules termed specific and nongenetic IAP-dependent protein erasers (SNIPERs) induced the ubiquitination and proteasomal degradation of ERα [103].

Recently, Xia X. et al. reported the role of deubiquitination in ERα stabilization in human breast tumor tissue [104]. They discovered that ubiquitin specific protease 7 (USP7) physically interacted with ERα, thereby mediating the deubiquitination and stabilization of ERα. USP7 inhibition led to apoptosis of ERα-positive breast cancer cells. The authors believe that targeting the USP7-ERα complex could be a potential strategy to treat ERα-positive breast cancer. However, the role of UPS in the regulation of ERα is beyond the control of protein content, since proteasome chymotrypsin-like activity is involved in the regulation of the expression of the gene encoding ERα (ESR1). The ESR1 gene, measuring 450,000 pairs of nucleotides, is regulated by seven different promoters that lead to the formation of various transcripts [105]. The evaluation of ESR1 mRNA levels by real-time quantitative PCR showed that the addition of bortezomib, a proteasome inhibitor, to the cell culture resulted in an 18% decrease in the level of ESR1 mRNA, but its combined use with estradiol, which also reduced the level of ESR1 mRNA, led to a 95% decrease in ESR1 mRNA expression relative to the control group. Powers G.L. et al. discovered that bortezomib induced a dramatic decrease in ERα mRNA in breast cancer cells, due to direct transcriptional inhibition and loss of RNA polymerase II recruitment on the ERα gene promoter [99]. The chronic inhibition of proteasomes (for 24 h or more) leads to an almost complete loss of ERα in cell culture [106]. The loss of ERα is the result of transcriptional repression of the ESR1 gene, as evidenced by a decrease in ESR1 mRNA. ESR1 mRNA levels decreased by 90% in several cell models, with a high expression of ERα (mammary gland, uterus, and pituitary gland), after the addition of bortezomib to cell cultures.

LMP2-associated proteasome subtype is required for ER-mediated gene transcription, both at initiation and elongation stages [107]. In these processes, LMP2 interacts directly with the SRCs. The recruitment of LMP2 by SRCs is necessary for cyclic association of ER-regulated transcription complexes on ER target genes. Besides, the proteasome subtype with the LMP2 subunit is able to promote estrogen-stimulated cell cycle progression. It is likely that the level of this proteasome subtype in a particular tissue could influence the content and/or the activity of specific proteins that are critical for proliferation [107]. Interestingly, proteasome inhibition leads to transcriptional elongation defects on estrogen target genes and to decreased chromatin dynamics overall [108]. There is evidence that GPER1, a membrane receptor, is degraded in two ways: after the internalization into endosomes, GPER1 is absorbed by lysosomes, but parts of the receptors are ubiquitinated before the internalization and transported to the Golgi complex, where the receptors are additionally phosphorylated and then destroyed by proteasomes. The use of the proteasome inhibitor MG132 reduced the degree of degradation of GPER1. It was shown that about 10% of GPER were cleaved in lysosomes and 90% in proteasomes [109].

The degradation of ER in proteasomes is of great importance under physiological conditions in target tissues, such as the mammary gland, endometrium and myometrium. In addition, estrogens affect the cardiovascular system, including having effects on vascular function, cardiomyocytes, and stem cell survival [110]. UPS influences the function of the cardiovascular system through ER by post-translational modification, ubiquitinating the receptors and signaling pathway components, as well as cleaving estrone receptors [110]. Estrogens play an important role in the regulation of bone metabolism, primarily through the membrane receptor GPER1, which is actively degraded by the proteasomes [111].

Importantly, the back-action of ERs on the proteasome pool was discovered. Our studies revealed the influence of the presence or absence of ERα on the expression of proteasomes in breast cancer tissue, with the use of a multiple analysis of generalized linear models, which allowed the detection of the relationships that were not detected by other statistical methods [112]. This fact indicates that the expression of proteasomes operates under an excessively complex mechanism of regulation, which can be completely understood only by using the techniques and analytical procedures normally utilized in multidimensional cognition [113]. We tested the effect of this factor, together with the tumor size factor on the expression of the 19S RC and immune proteasomes containing LMP2 and/or LMP7 subunits in the primary tumor and adjacent conditionally normal tissue. The expressions of the LMP2 subunit in the tumor, LMP7 subunit in the adjacent tissue, and 19S RC, both in the tumor and adjacent tissue, were found to be dependent on the presence or absence of ERα. In the absence of ERα, the studied proteasome parameters increased significantly with the increase of the tumor size. In tumor cells (not in stromal cells), the main quantities of the LMP2 subunit and 19S RC were fixed by immunohistochemistry. In contrast, in the presence of ERα, all of these proteasome parameters tended to decrease with the increase of the tumor size.

**Figure 3:**
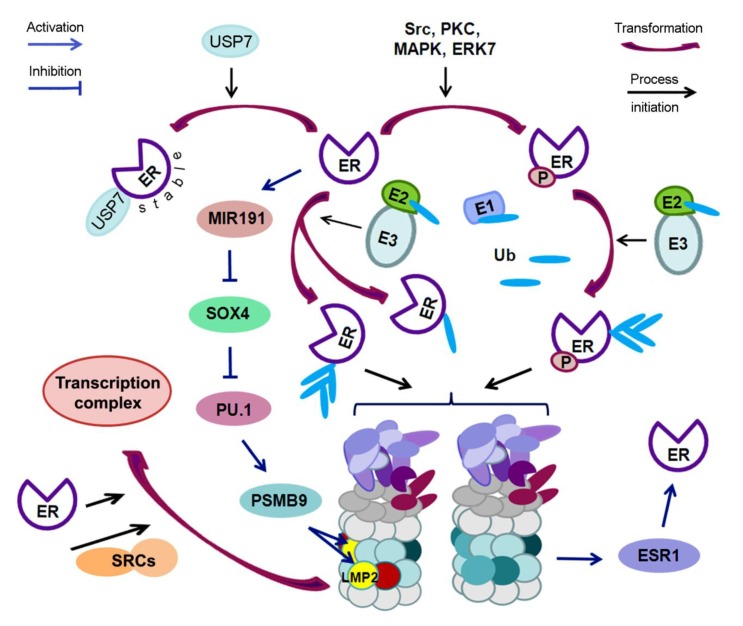
Scheme of the mutual regulation of estrogen receptors and ubiquitin proteasome system. ER—estrogen receptor; P—phosphate group; Src, PKC, MAPK, ERK7—protein kinases; USP7—ubiquitin specific protease 7; E1—ubiquitin-activating enzyme; E2—ubiquitin-conjugating enzyme; E3—ubiquitin protein ligase; Ub—ubiquitin; ESR1—gene encoding ERα; SRCs—steroid receptor coactivators; MIR191—miRNA 191; SOX4, PU.1—transcription factors; LMP2—large multifunctional peptidase 2 (immune proteasome subunit); PSMB9—gene of proteasome subunit beta type 9 (LMP2).

The revealed complex dependence of the LMP2 subunit expression in the tumor on the simultaneous effect of ERα expression and other factors raises the question of how ERα can affect LMP2 expression. This can be explained by the functioning of ERα as a factor that indirectly regulates the LMP2 expression through the participation of miRNAs (MIR), and some transcription factors. Thus, ERα induces the expression of MIR191 [114]. In turn, MIR191 inhibits the expression of a number of genes, including gene of SRY-related HMG-box transcription factor 4 SOX4, in breast carcinoma [115]. The transcription factor SOX4 can affect the mRNA level of the transcription factor PU.1 [116], which directly transactivates the proteasome subunit beta type 9 PSMB9 (LMP2) promoter [117]. The relationship between ERs and UPS is schematically presented in Figure 3.

Interestingly, the detected dependence of proteasome expression in the tumor on the presence or absence of ERα touches the proteasome subtype containing the proteolytic subunit LMP2 (but not LMP7). Obviously, this proteasome subtype in tumor cells, as well as in allograft cells, is involved in the development of immunological tolerance [87].

According to our results, the LMP2 subunit and 19S RC may be promising targets for the treatment of breast cancer. Indeed, antitumor compositions that violate the functions of the 19S RC were effective against mammary adenocarcinoma Ca 755 in mice [118]. Similar medicinal substances can be relevant for the treatment of breast cancer in the absence of ER, when the therapy that blocks ER signaling is useless. A flexible approach to the medical treatment of cancer that is based on different targets and influences different regulation systems—i.e., an approach grounded on the results of continuous investigations—is preferable to the usual multiplication of drugs aimed at a single mechanism, and which differ very slightly from each other in their structure (e.g., by one structural group or ligand). The latter approach is normally applied by some Western pharmaceutical companies. That may be an indication of the extensive development of cancer therapy, with the process, among many others, unfortunately taking place in different areas of current biotechnological and pharmaceutical engineering [119].

The close relationship between ERα and proteasomes in malignant tumor cells was also confirmed by Thaler S. et al., who showed that bortezomib inhibited ERα expression and induced the death of ERα^+^ breast cancer cells [120]. A study of breast cancer tissue revealed the positive correlation between the expression of proteasome activator REGγ and ERα status [121]. On the whole, the development of estrogen-dependent malignant tumors is accompanied by an increase in the chymotrypsin-like activity of the proteasomes and a change in their subunit composition [122,123,124]. The use of proteasome inhibitors, together with antiestrogens, against the ER-positive culture of breast cancer leads to a significantly increased antitumor effect, compared to the action of these drugs in a single mode [125]. Inhibitors of deubiquitinases Usp14 and Rpn11 suppress the growth of estrogen-positive breast cancer cells, which is accompanied by cell cycle arrest, apoptosis induction, and a decrease in ERα content [126]. Thus, the study of impaired ER and UPS regulation in pathological conditions is important for the understanding the mechanisms of disease development and the search for new therapeutic approaches to their treatment.

## 5. Final Remarks

ERs have a significant effect on normal and pathological cellular events, due to their ability not only to activate gene expression, but also to interact with signaling pathways. UPS, the regulator of multiple cellular processes, can change the content and functional activity of ERs in different ways. In turn, ERs can affect UPS; in particular, proteasome subunit composition. In the present review, we sought to provide the current information concerning not only the properties and mechanisms of action of ERs and UPS, but also the ways in which they interact in normal and some pathological conditions. Understanding the complex mechanisms underlying the mutual regulation of ERs and UPS promises much regarding the development of new therapeutic agents that can make a significant contribution to the treatment of diseases associated with the impaired function of these crucial biomolecules.
